# Differential Responses to Virus Challenge of Laboratory and Wild Accessions of Australian Species of *Nicotiana*, and Comparative Analysis of *RDR1* Gene Sequences

**DOI:** 10.1371/journal.pone.0121787

**Published:** 2015-03-30

**Authors:** Stephen J. Wylie, Chao Zhang, Vicki Long, Marilyn J. Roossinck, Shu Hui Koh, Michael G. K. Jones, Sadia Iqbal, Hua Li

**Affiliations:** 1 Plant Biotechnology Research Group-Virology, Western Australian State Agricultural Biotechnology Centre, School of Veterinary and Life Sciences, Murdoch University, Perth, Western Australia, Australia; 2 College of Plant Protection, Northwest Agriculture and Forestry University, Yangling, Shaanxi Province, China; 3 Astron Environmental Services, Karratha, Western Australia, Australia; 4 Plant Biotechnology Research Group—Pests, Western Australian State Agricultural Biotechnology Centre, School of Veterinary and Life Sciences, Murdoch University, Perth, Western Australia, Australia; 5 Departments of Plant Pathology and Environmental Microbiology, and Biology, Pennsylvania State University, University Park, Pennsylvania, United States of America; University of Basel, SWITZERLAND

## Abstract

*Nicotiana benthamiana* is a model plant utilised internationally in plant virology because of its apparent hyper-susceptibility to virus infection. Previously, others showed that all laboratory accessions of *N*. *benthamiana* have a very narrow genetic basis, probably originating from a single source. It is unknown if responses to virus infection exhibited by the laboratory accession are typical of the species as a whole. To test this, 23 accessions of *N*. *benthamiana* were collected from wild populations and challenged with one to four viruses. Additionally, accessions of 21 other *Nicotiana* species and subspecies from Australia, one from Peru and one from Namibia were tested for susceptibility to the viruses, and for the presence of a mutated RNA-dependent RNA polymerase I allele (*Nb-RDR1m*) described previously from a laboratory accession of *N*. *benthamiana*. All Australian *Nicotiana* accessions tested were susceptible to virus infections, although there was symptom variability within and between species. The most striking difference was that plants of a laboratory accession of *N*. *benthamiana* (RA-4) exhibited hypersensitivity to Yellow tailflower mild mottle tobamovirus infection and died, whereas plants of wild *N*. *benthamiana* accessions responded with non-necrotic symptoms. Plants of certain *N*. *occidentalis* accessions also exhibited initial hypersensitivity to Yellow tailflower mild mottle virus resembling that of *N*. *benthamiana* RA-4 plants, but later recovered. The mutant *Nb-RDR1m* allele was identified from *N*. *benthamiana* RA-4 but not from any of 51 other *Nicotiana* accessions, including wild accessions of *N*. *benthamiana*, demonstrating that the accession of *N*. *benthamiana* used widely in laboratories is unusual.

## Introduction

The genus *Nicotiana*, family Solanaceae, comprises 64 described species, the best known and perhaps most infamous of which are *N*. *tabacum* and *N*. *rustica* from the Americas, which form the basis of the tobacco industry. The genus is divided into 13 sections, the largest of which is *Suaveolentes*. *Nicotiana* section *Suaveolentes* holds 30 species, 27 of which are endemic to Australia, two to the Pacific Islands and one to Africa [1, 2]. The species within *Suavalentes* best known to the plant science community is *N*. *benthamiana*, an allotetraploid thought to originate from diploid parents [3, 4]. *N*. *benthamiana* occurs sporadically across approximately 3000 kilometres of northern Australia from longitudes 114°E to 140°E and between latitudes 14°S and 26°S. Like *Nicotiana* species from the Americas, the Australian members of the genus contain nicotine and other alkaloids that stimulate the human central nervous system, and these compounds made members of the genus important to the Aboriginal peoples of Australia [5]. From the leaves of various *Nicotiana* species and the related genus *Duboisia* they made *Pituri* (also known as *Tjuntiwari*, *Muntju*, *Pinkaraangu*, *Mingulba* and other names) [5, 6], a product made of dried or baked leaves and wood ash to form a wad that was held in the mouth between the teeth and gums [5, 7, 8]. *N*. *benthamiana* was not the most favoured species for making *Pituri*, but it was used when more desirable species were unavailable [8].

*N*. *benthamiana* is valued today not only because of its susceptibility to over 500 plant viruses [9], but also because of its susceptibility to infection by bacteria, fungi, oomycetes and nematodes [10, 11, 12, 13]. It is used for transient expression of transgenes through agroinfiltration, where *Agrobacterium tumefaciens* harbouring a T-DNA plasmid is introduced locally into a leaf. Transient local expression of genes from the T-DNA region by the plant enables studies in protein expression and regulation in the infiltrated leaf without the need to express transgenes stably in a whole plant [9, 12, 14, 15, 16]. As a reflection of its importance in the plant sciences, two draft genome sequences and transcriptome sequences of *N*. *benthamiana* have been released [16, 17,18].

The evolutionary basis of the apparent hyper-susceptibility to viruses by *N*. *benthamiana* is unclear. Being highly susceptible to many viruses would seem at first glance to place the species on a fast track to extinction, but recent research has shown that viruses with long associations with wild plants are seldom severe pathogens [19]. Under experimental conditions, only a minority of plant viruses actually kill *N*. *benthamiana* plants; most infect with mild to moderate symptoms and often the plant is able to reproduce. The climate in which *N*. *benthamiana* grows may offer protection from severe virus-induced symptoms. *N*. *benthamiana* grows in Australia’s north where daytime temperatures can reach above 40°C (>104°F). The earliest report we could find in the scientific literature describing *N*. *benthamiana* and responses to virus infection showed how high growing temperatures ameliorated symptoms of Tobacco mosaic virus (TMV) infection [20]. Another suggestion is that wild populations of *N*. *benthamiana* live in zones relatively free of plant virus incidence, making resistance to viruses an unnecessary trait. Although virus surveys of wild plants have not been undertaken in most of the natural range of *N*. *benthamiana*, there is no reason to suppose that viruses are not present in the flora there. Further south in Western Australia, new viruses are regularly encountered in the indigenous flora [21, 22, 23, 24, 25, 26, 27, 28, 29, 30]. If natural populations of *N*. *benthamiana* are indeed highly susceptible to virus infection, might infection confer an evolutionary advantage under certain environmental conditions and/or at some stages of the life cycle? In the controlled conditions of the laboratory, *N*. *benthamiana* plants infected with Cucumber mosaic virus (CMV) lived about 20% longer under drought conditions than did uninfected plants [31], probably because virus-infected plants accumulate glycol, myo-inositol and other water stress-related protectants [32]. If the same phenomenon occurs amongst wild plants living in regions of variable water availability and seasonally arid conditions, such as occur in the areas that *N*. *benthamiana* naturally inhabits, it is conceivable that sub-lethal virus infections in later stages of the life cycle may enable plants to tolerate drought longer than uninfected plants, perhaps long enough to complete the life cycle.

A possible genetic basis to virus susceptibility in *N*. *benthamiana* was provided by Yang *et al*. [33] who identified that the RNA-dependent RNA polymerase I (*Nb-RDR1*) involved in small interfering RNA synthesis and virus resistance, contained a 72 nucleotide insertion mutation that introduced tandem stop codons. The mutant allele was referred to as *Nb-RDR1m* (*Nicotiana benthamiana* RNA-dependent RNA polymerase I mutant).

Using AFLP analysis Goodin *et al*. [12] showed that *N*. *benthamiana* accessions used in laboratories have a very narrow genetic basis. They named the five accessions gathered from laboratories around the world Research Accession (RA) 1–5 and concluded they were probably all derived from a single source. The source of the original laboratory accessions is not published. In collections of wild *N*. *benthamiana* lines held by Australian herbaria there exist specimens collected from different habitats, and these show variation in plant size and structure, leaf and flower shape, and other traits [12].

Other Australian *Nicotiana* species have been utilized by science to a much lesser extent than has *N*. *benthamiana*. The best known is *N*. *occidentalis*, where several accessions, for example B37 (also known as 37B), P-1, P12, and N1, are identified and have been used in virus-related studies [34, 35, 36, 37, 38]. These accession codes probably refer to members of *N*. *occidentalis* ssp *obliqua*, the most widespread subspecies in Australia. Although *N*. *hesperis* was described in 1960 [39] and accession 67A of this species has been cited in scientific reports up until the present day [38, 40, 41, 42], *N*. *hesperis* has not existed as a species since 1981 when it was reclassified as *N*. *occidentalis* subspecies *hesperis* [43]. This subspecies has a limited natural distribution, being restricted to northern coastal regions of Western Australia. We could find no records of scientific use of the third subspecies, *N*. *occidentalis* ssp *occidentalis*, also restricted to northern coastal and island sites in Western Australia. Despite recommendations that *N*. *cavicola*, *N*. *rosulata*, *N*. *ingulba* (syn *N*. *rosulata* ssp *ingulba*), and *N*. *rotundifolia* are useful experimental hosts in the diagnosis of plant viruses [10], apparently none of these Australian species have been widely adopted for this purpose. The reason is unclear, but perhaps it is because of limited availability of their seed or because of the broader availability of *N*. *benthamiana*, *N*. *occidentalis* and non-Australian species such as *N*. *tabacum*, *N*. *clevelandii*, and *N*. *glutinosa* [44].

Here, we tested responses of laboratory and wild accessions of *N*. *benthamiana*, accessions of the three subspecies of *N*. *occidentalis*, accessions of 19 other Australian *Nicotiana* species, a South American *Nicotiana* species, and the sole African *Nicotiana* species to plant viruses. Partial *RDR1* gene sequences were obtained from some accessions, and we speculate further on the role of this gene in virus susceptibility and symptom development.

## Materials and Methods

### Plants

**Fig 1:**
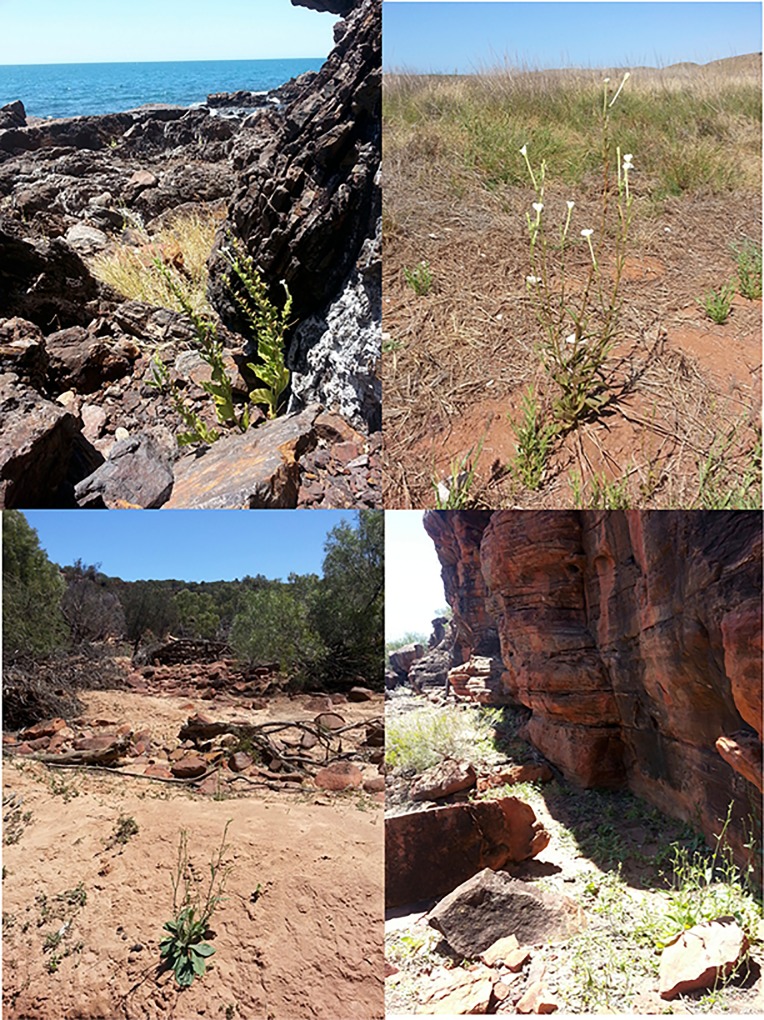
*Nicotiana* plants in their natural habitats in Australia. Top left, a *N*. *benthamiana* plant growing amongst rocks beside the Indian Ocean in the Pilbara region. Top right, a *N*. *occidentalis* ssp *occidentalis* plant growing on coastal spinifex grasslands near Roeburn. Lower left, a *N*. *rotundifolia* plant on a dry riverbed in the Murchison Region. Lower right, a group of *N*. *occidentalis* ssp *obliqua* plants growing at the base of a rock face in the Pilbara region.

Plants were collected under licence and a Regulation 4 Authority issued by the Western Australian Department of Parks and Wildlife. Eighteen accessions of *N*. *benthamiana*, 26 accessions of *N*. *occidentalis*, including three of subspecies *hesperis*, 14 of subspecies *obliqua*, four of subspecies *occidentalis*, two *N*. *occidentalis* accessions for which the subspecies was not determined, three accessions *N*. *rotundifolia*, two accessions of *N*. *heterantha*, one accession of *N*. *umbratica*, and three *Nicotiana* accessions that were not identified to the species level were collected from multiple wild populations located in northern Western Australia (Table 1, Fig 1). Accessions referred to as ‘Seed Lines’ (SL) of 20 other *Nicotiana* species indigenous to Australia and one from Namibia (*N*. *Africana*, section Suaveolentes) were kindly provided as seed by Dr Edward Newbigin, University of Melbourne (Table 1). Plants of a laboratory accession of *N*. *benthamiana*, which we designated RA-4 after Goodin *et al*. [12], *N*. *glutinosa* (section *Undulatae*, naturally occuring from Bolivia to Peru), and *Chenopodium amaranticolor* (local lesion host native to South America) were already available. All plants were grown in a rotted bark and sand mix to which 5 g each of lime and dolomite and 40 g of slow release NPK fertiliser was added per 40 litres of potting mix. When the germination rate was low or uneven, seed was soaked overnight at room temperature in a solution of 100 mM gibberellic acid (GA_4_) to stimulate germination [45].

**Table 1:** Accessions used, showing species and their origins.

Species	Accession/Seed Line	Origin (if known)^a^	Habitat (if known)	Year collected (if known)	Reference
*Chenopodium amaranticolor*					
*C*. *quinoa*		Peruvian Andes	Montane		
*Nicotiana africana*	SL6	Namibia		1983	[46]
*N*. *amplexicaulis*	SL7	Carnarvon Range, QLD		1957	[46]
*N*. *benthamiana*	17.19C	Cleaverville Cove, WA	Coastal cliffs	2013	This study
*N*. *benthamiana*	17.23	Hammersley Range, WA	Cave entrance, inland riverbed	2013	This study
*N*. *benthamiana*	17.24	Millsteam-Chichester National Park, WA	Cliffs, inland riverbed	2013	This study
*N*. *benthamiana*	17.26	Millsteam-Chichester National Park, WA	Cliffs, inland riverbed	2013	This study
*N*. *benthamiana*	17.27	Millsteam-Chichester National Park, WA	Inland riverbed	2013	This study
*N*. *benthamiana*	17.28	Millsteam-Chichester National Park, WA	Inland riverbed	2013	This study
*N*. *benthamiana*	Cl-1	Cleaverville Cove, WA	Coastal cliffs	2013	
*N*. *benthamiana*	HCK-1	Honeymoon Cove, WA	Coastal cliffs	2013	This study
*N*. *benthamiana*	HCK-3	Honeymoon Cove, WA	Coastal cliffs	2013	This study
*N*. *benthamiana*	KL-1	Kalamina Gorge, Karijini National Park, WA	Cliffs, inland riverbed	2013	This study
*N*. *benthamiana*	KL-2	Kalamina Gorge, Karijini National Park, WA	Cliffs, inland riverbed	2013	This study
*N*. *benthamiana*	KL-3	Kalamina Gorge, Karijini National Park, WA	Cliffs, inland riverbed	2013	This study
*N*. *benthamiana*	KL-4	Kalamina Gorge, Karijini National Park, WA	Cliffs, inland riverbed	2013	This study
*N*. *benthamiana*	KL-5	Kalamina Gorge, Karijini National Park, WA	Cliffs, inland riverbed	2013	This study
*N*. *benthamiana*	Kx-1	Knox Gorge, Karijini National Park, WA	Cliffs, inland riverbed	2013	This study
*N*. *benthamiana*	MtA-3	Mt Augustus region, WA	Rock overhang, inland riverbed	2013	This study
*N*. *benthamiana*	MtA-5	Mt Augustus region, WA	Rock overhang, inland riverbed	2013	This study
*N*. *benthamiana*	MtA-6	Mt Augustus region, WA	Rock overhang, inland riverbed	2013	This study
*N*. *benthamiana*	MtA-7	Mt Augustus region, WA	Rock overhang, inland riverbed	2013	This study
*N*. *benthamiana*	PPM-1	Millsteam-Chichester National Park, WA	Cliffs, inland riverbed	2013	This study
*N*. *benthamiana*	PPM-2	Millsteam-Chichester National Park, WA	Cliffs, inland riverbed	2013	This study
*N*. *benthamiana*	RA-4				
*N*. *benthamiana*	VL552B2.1	Cleaverville Cove, WA	Coastal cliffs	2012	This study
*N*. *benthamiana*	VL552B2.2	Cleaverville Cove, WA	Coastal cliffs	2012	This study
*N*. *cavicola*	SL9	Meekathara, WA		1956	[46]
*N*. *excelsior*	SL11			1998/99	[46]
*N*. *forsteri (syn N*. *debneyi)*	SL5			2003	[46]
*N*. *glutinosa*					[46]
*N*. *goodspeedii*	SL13	Port Augusta, SA		1955	[46]
*N*. *gossei*	SL14	Henbury, NT			[46]
*N*. *heterantha*	Ft-2	Fortesque River, WA	Inland riverbed	2013	This study
*N*. *heterantha*	Ft-3	Fortesque River, WA	Inland riverbed	2013	This study
*N*. *heterantha*	SL33			2005	[46]
*N*. *maritima*	SL35				[46]
*N*. *megalosiphon*	SL1				[46]
*N*. *occidentalis ssp (or N*. *suaveolens)*	SL15				[46]
*N*. *occidentalis*	Cl-1	Cleaverville Plain, WA	Inland plain 2 km from coast	2013	This study
*N*. *occidentalis ssp hesperis*	Nt-1	Ashburton River, Nanutarra, WA	Inland, dry riverbed	2013	This study
*N*. *occidentalis ssp hesperis*	Nt-4	Ashburton River, Nanutarra, WA	Inland, dry riverbed	2013	This study
*N*. *occidentalis ssp hesperis*	Nt-5	Ashburton River, Nanutarra, WA	Inland, dry riverbed	2013	This study
*N*. *occidentalis ssp obliqua*	Ft-1	Fortesque River, WA	Verge, public carpark	2013	This study
*N*. *occidentalis ssp obliqua*	MtA-10	Mt Augustus region, WA	Inland plain	2013	This study
*N*. *occidentalis ssp obliqua*	MtA-11	Mt Augustus region, WA	Inland plain	2013	This study
*N*. *occidentalis ssp obliqua*	MtA-12	Mt Augustus region, WA	Inland plain	2013	This study
*N*. *occidentalis ssp obliqua*	MtA-4	Mt Augustus region, WA	Inland plain	2013	This study
*N*. *occidentalis ssp obliqua*	MtA-8	Mt Augustus region, WA	Inland plain	2013	This study
*N*. *occidentalis ssp obliqua*	MtA-9	Mt Augustus region, WA	Inland plain	2013	This study
*N*. *occidentalis ssp obliqua*	SL17	Earsbiddy Hills, WA		1956	[46]
*N*. *occidentalis ssp obliqua*	UK-1	Tom Price region, WA	Inland plain	2013	This study
*N*. *occidentalis ssp obliqua*	UK-2	Tom Price region	Inland plain	2013	This study
*N*. *occidentalis ssp obliqua*	UK-3	Tom Price region	Inland plain	2013	This study
*N*. *occidentalis ssp obliqua*	UK-4	Tom Price region	Inland plain	2013	This study
*N*. *occidentalis ssp obliqua*	VL552B1.1	Varanus Island, Lowendal Island group, WA	Coastal	2013	This study
*N*. *occidentalis ssp occidentalia*	CB-1	Coral Bay, WA	Road verge, coastal township	2013	This study
*N*. *occidentalis ssp occidentalis*	BC-1	Boundary Creek, Exmouth region, WA	Coastal dry riverbed	2013	This study
*N*. *occidentalis ssp occidentalis*	Br-1	Bridled Island, Lowendal Island group, WA	Coastal	2013	This study
*N*. *occidentalis ssp occidentalis*	SC-2A	Shothole Canyon, Cape Range National Park, WA	Inland, cliffs	2013	This study
*N*. *occidentalis ssp occidentalis*	TB-1	Turquoise Bay, Cape Range National Park, WA	Coastal	2013	This study
*N*. *rosulata ssp ingulba*	SL18	Curtain Springs, NT		1953	[46]
*N*. *rosulata ssp rosulata*	SL51				[46]
*N*. *rotundifolia*	SL20				[46]
*N*. *rotundifolia*	RG-1	Murchison River, WA	Inland riverbed	2013	This study
*N*. *rotundifolia*	HH-1	Murchison River, WA	Inland riverbed	2013	This study
*N*. *rotundifolia*	KB-1	Kalbarri, WA	Road verge in township	2013	This study
*N*. *simulans*	SL19	Wiluna, WA			[46]
*N*. *simulans*	BrH-1	Sampson Point, WA	Road verge	2013	This study
*N*. *simulans*	SL29	Ayr, QLD		1955	[46]
*N*. *suaveolens*	SL24	Flinders Peak, VIC		2003	[46]
*N*. *truncata*	SL44	Fish Hole Creek, SA		2005	[46]
*N*. *umbratica*	Wea-1	Weano Gorge, Karijini National Park, WA	Inland riverbed	2013	This study
*Nicotiana sp*	MtG-2	Mount Gould, WA	Inland, dry riverbed	2013	This study
*Nicotiana sp*	MtG-4	Mount Gould, WA	Inland, dry riverbed	2013	This study
*Nicotiana sp 'Corunna'*	SL23	Corunna, WA		2005	[46]

^a^ NT, Northern Territory; QLD, Queensland; SA, South Australia; VIC, Victoria; WA, Western Australia

### Viruses

Virus isolates used to challenge plants.

1. Yellow tailflower mild mottle virus isolate Cervantes (YTMMV, genus *Tobamovirus*, GenBank accession KF495565) was originally isolated from a wild plant of Yellow Tailflower (*Anthocercis littoria*, family *Solanaceae*) at Cervantes, Western Australia [30]. The plant was collected under a flora permit issued by the Western Australian Department of Parks and Wildlife.
2. Bean yellow mosaic virus isolate SW3.2 (BYMV, genus *Potyvirus*, GenBank accession JX156423) was originally isolated from a wild donkey orchid plant (*Diuris longifolia*, family Orchidaceae) at Brookton, Western Australia [29]. The plant was collected under a flora permit issued by the Western Australian Department of Parks and Wildlife.
3. Cucumber mosaic virus isolate SW-11 (CMV, subgroup II, genus *Cucumovirus*, GenBank accessions KM434204, KM434205, and KM434206) was isolated from a plant of *Cymbidium* species (family *Orchidaceae*) growing on private property belonging to co-author MGK Jones in Perth, Western Australia, with his permission.
4. Tomato spotted wilt virus isolate WA-7 (TSWV, genus *Tospovirus*, GenBank accessions KM365064, KM365065, and KM365066) was originally isolated from a seedling of tomato cv Money Maker (*Solanum lycopersicum*, family *Solanaceae*) purchased from a garden supply store in Perth, Western Australia. No specific permissions were required to collect this plant.

Virus isolates of BYMV, CMV and TSWV were maintained in plants of *N*. *benthamiana* RA-4 where they were subcultured every 110–140 days. The genome sequences of each virus isolate were fully or largely determined from double-stranded RNA enriched fractions from the systemic host, *N*. *benthamiana*. For YTMMV, leaf sap from the original wild host *Anthocercis littoria* was used to inoculate a plant of *N*. *benthamiana* RA-4, and the isolate was subcultured every 20–30 days to a new plant before the previous host died.

### Inoculation of *Nicotiana* plants

After germination, seedlings were grown to the 4-leaf stage before they were subjected to either mock inoculation with 0.1M phosphate buffer (pH7) and diatomaceous earth (Sigma Corp.) or inoculation as above with the addition of macerated leaf material from a virus-infected plant. Five to ten plants of each accession were used for each treatment, and an equal number were used for mock inoculations. Treated plants were grown in climate-controlled, insect-free greenhouses where they were provided with optimal growing conditions (22°C day and 17°C night temperatures, daily watering, weekly nutrient feeds).

### Symptom category index and statistical analysis

Symptom development was recorded on infected plants every two to five days until 35 days post-inoculation (dpi). All plants were tested for the presence of systemic spread of virus in uninoculated young leaves at 35 dpi using virus-specific primers (S1 Table) in RT-PCR assays (below).

**Fig 2:**
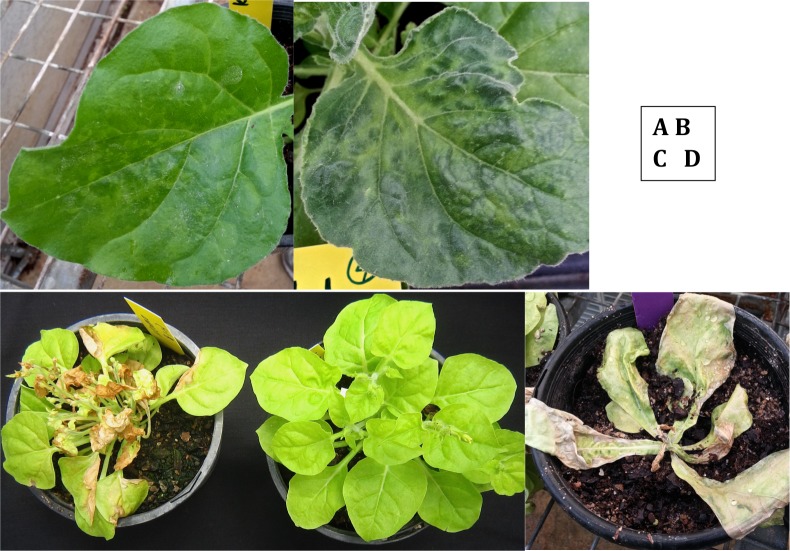
Symptom indices. A: Index 2, mild symptoms including faint mosaic, little stunting or leaf distortion. Example given is from *N*. *benthamiana* accession KL-1 infected with BYMV. B: Index 3, moderate symptoms of infection including strong mosaic and some leaf distortion and plant stunting. Example given is from *N*. *benthamiana* accession KL-1 infected with YTMMV. C: Index 4, severe necrosis affecting most of the plant. No flowers. Example given is from *N*. *umbratica* accession Wea-1 infected with YTMMV (left). Plant on the right is uninfected. D: Index 5, whole plant is dead.

Plant symptoms and infections were also scored 35 dpi using a simple assessment of systemic infection and symptom severity indices as follows (Fig 2):

0. No systemic infection detected. Local necrotic lesions (NL) may present on inoculated leaves.
1. Systemic spread confirmed by RT-PCR. No visible symptoms of infection observed.
2. Systemic spread confirmed by RT-PCR. Mild symptoms of chlorosis, mosaic and/or leaf deformation evident. Slight stunting may occur. Ring patterns (rings) or small necrotic lesions (NL) sometimes visible.
3. Systemic spread confirmed by RT-PCR. Moderate symptoms of chlorosis, mosaic and/or leaf deformation. Moderate to significant stunting of growth and small necrotic lesions may be present. Flowers usually present.
4. Systemic spread confirmed by RT-PCR. Large necrotic lesions on leaf/stem tissue that affect more than half of the plant. Severe stunting. Plant remained alive but apparently not actively growing. No flowers present.
5. Systemic spread confirmed by RT-PCR. The plant was dead by 35 dpi

Statistical analysis of variance of symptoms within and between plant accessions and viruses was done using the software package IBM SPSS Statistics 21.

### Confirmation of infection status

Inoculated plants were screened at 35 dpi for presence of the virus using virus-specific primers (S1 Table) in RT-PCR assays. The MyTaq One-Step RT-PCR kit (Bioline) was used to synthesise cDNA and amplify fragments of virus genomes in the presence of virus-specific forward and reverse primers from total RNA extracted from plants using the RNeasy Plant Mini kit (Qiagen) or a dsRNA enrichment method [47] modified by replacing Whatman CF11 cellulose powder with Machery Nagel MN100 cellulose powder. Virus-specific primers were used for YTMMV [30], BYMV [48], CMV [49], and TSWV [50] (S1 Table). Subsequent Sanger sequencing of amplicons was done using the amplification primers and with BigDye terminator V3.1 chemistry, and analysed with an Applied Biosystems/Hitachi 3730 DNA Analyzer. Sequences were edited manually in FinchTV (Geospiza) and aligned using ClustalW [51].

### *RDR1* gene sequencing

DNA was extracted from 100 mg young leaf tissue using DNeasy Plant Mini kit columns (Qiagen). Primers were designed to flank the region of the *Nb-RDR1m* allele 72 nt insertion mutation [33]. Three sets of primers were used. Primers RP1 and RP2 [33], which generated an amplicon of approximately 327 nt or 255 nt, depending on the presence of the insertion mutation. Primers RP120614 and RP220614 generated amplicons of 351 nt or 279 nt, and primers RP1new and RP2new generated amplicons of 389 nt or 317 nt (S2 Table). Amplification was done using GoTaq DNA Polymerase (Promega Corp). The parameters used for subsequent Sanger sequencing of amplicons were as described above.

## Results

Significant differences (p<0.05) between plant responses to virus infection were recorded (Table 2, S2 Table, S3 Table, Fig 2, S1 Fig.).

**Table 2:** Mean symptom severity index (Standard Deviation above 0 in parenthesis) of virus infection on plants expressed 35 dpi.

Species^a^ and subspecies (where known)	Accession/Seed line	YTMMV	BYMV	CMV	TSWV	GenBank Accession *RDR1* (partial)	indel present Y/N
			Symptom index^b^				
*Chenopodium amaranticolor*		0.0 (NL)	0.0 (NL)	0.0(NL)	0.0(NL)		
*C*. *quinoa*		0.0 (NL)	0.0 (NL)	0.0(NL)	0.0(NL)		
*Nicotiana africana*	SL6	2.0	1.4(0.55)	1.0	1.0		
*N*. *amplexicaulis*	SL7		1.0	2.0	2.0	KM411324	N
*N*. *benthamiana*	17.19C	2.6(0.54)				KM411316	N
*N*. *benthamiana*	17.23	3.0				KM411311	N
*N*. *benthamiana*	17.24	3.0				KM411313	N
*N*. *benthamiana*	17.26	3.0				KM411325	N
*N*. *benthamiana*	17.27	3.0				KM411314	N
*N*. *benthamiana*	17.28	3.0				KM411315	N
*N*. *benthamiana*	Cl-1		2.0		3.0		
*N*. *benthamiana*	HCK-1	3.8(0.45)					
*N*. *benthamiana*	HCK-3	3.8(0.45)					
*N*. *benthamiana*	KL-1	3.0	2.2(0.45)	2.8(0.45)	3	KM411351	N
*N*. *benthamiana*	KL-2	3.0	2.2(0.45)	2.6(0.55)	3.2(0.45)	KM411352	N
*N*. *benthamiana*	KL-3		2.0	2.0	3.0	KM411349	N
*N*. *benthamiana*	KL-4		2.0	2.0	3.0	KM411353	N
*N*. *benthamiana*	KL-5		2.0		3.0	KM411319	N
*N*. *benthamiana*	Kx-1	3.0	3 (NL)	3.0	3.0	KM411307	N
*N*. *benthamiana*	MtA-3	3.0				KM411321	N
*N*. *benthamiana*	MtA-5	3.0				KM411312	N
*N*. *benthamiana*	MtA-6	3.0					
*N*. *benthamiana*	MtA-7	3.0				KM411304	N
*N*. *benthamiana*	PPM-1	3.0					
*N*. *benthamiana*	PPM-2	3.0				KM411341	N
*N*. *benthamiana*	RA-4	5.0	3.0	3.0	3.0	KM411308	Y
*N*. *benthamiana*	VL552B2.1	3.0	2.0	2.0	3.0	KM411346	N
*N*. *benthamiana*	VL552B2.2	3.0	2.0		3.0		
*N*. *cavicola*	SL9	5.0	3.8(0.45)	2.0	4.2(0.45)	KM411326	N
*N*. *excelsior*	SL11	4.6(0.55)	1.1	1.0	2.4(0.55)		
*N*. *forsteri*	SL5		1.0	1.0	3.0	KM411327	N
*N*. *glutinosa*		0.0 (NL)	0.0	0.0	0.0(NL)	KM411328	N
*N*. *goodspeedii*	SL13		4.0	3.0	3.0		
*N*. *gossei*	SL14		1.0	1.0	3.0	KM411329	N
*N*. *heterantha*	Ft-2	2.4(0.55)					
*N*. *heterantha*	Ft-3	2.2(0.45)				KM411310	N
*N*. *heterantha*	SL33	1.8(0.45)	4.2(0.55)	4.2(0.45)	3.4(0.55)		
*N*. *maritima*	SL35		3.0	3.0	3.0		
*N*. *megalosiphon*	SL1		2.0 (NL)	1.0	5.0	KM411330	N
*N*. *occidentalis hesperis (or N*. *suaveolens)*	SL15		2.0	1.0	4.0		
*N*. *occidentalis*	Cl-1	4.4(0.55)				KM411318	N
*N*. *occidentalis* ssp *hesperis*	Nt-1		4.0 (NL)	5.0 (NL)	5.0(NL)		
*N*. *occidentalis* ssp *hesperis*	Nt-4		4.0 (NL)	3.0 (NL)	4.0(NL)		
*N*. *occidentalis* ssp *hesperis*	Nt-5		4.0 (NL)	3.0 (NL)	5.0(NL)		
*N*. *occidentalis* ssp *obliqua*	Ft-1	4.4(0.55)					
*N*. *occidentalis* ssp *obliqua*	MtA-10	5.0				KM411322	N
*N*. *occidentalis* ssp *obliqua*	MtA-11	4.8(0.45)				KM411309	N
*N*. *occidentalis* ssp *obliqua*	MtA-12	4.8(0.45)				KM411323	N
*N*. *occidentalis* ssp *obliqua*	MtA-4	5.0				KM411332	N
*N*. *occidentalis* ssp *obliqua*	MtA-8	5.0					
*N*. *occidentalis* ssp *obliqua*	MtA-9	4.4(0.55)				KM411331	N
*N*. *occidentalis* ssp *obliqua*	SL17	5.0	4.0	1.0	4.0		
*N*. *occidentalis* ssp *obliqua*	UK-1	4.8(0.45)				KM411344	N
*N*. *occidentalis* ssp *obliqua*	UK-2	5.0				KM411343	N
*N*. *occidentalis* ssp *obliqua*	UK-3	4.6(0.55)					
*N*. *occidentalis* ssp *obliqua*	UK-4	5.0				KM411345	N
*N*. *occidentalis* ssp *obliqua*	VL552B1.1	5.0	2.0	2.0	5.0	KM411337	N
*N*. *occidentalis ssp occidentalis*	CB-1	3.8(0.45)				KM411317	N
*N*. *occidentalis* ssp *occidentalis*	BC-1	5.0				KM411334	N
*N*. *occidentalis* ssp *occidentalis*	Br-1	5.0				KM411320	N
*N*. *occidentalis* ssp *occidentalis*	SC-2A	4.8(0.45)				KM411335	N
*N*. *occidentalis* ssp *occidentalis*	TB-1	5.0				KM411336	N
*N*. *rosulata* ssp *ingulba*	SL18	5.0	1.4(0.45)	1.0	4.4(0.55)	KM411338	N
*N*. *rosulata* ssp *rosulata*	SL51	4.8(0.45)				KM411339	N
*N*. *rotundifolia*	SL20		1	1.0	4.0	KM411305	N
*N*. *rotundifolia*	HH-1	5.0				KM411347	N
*N*. *rotundifolia*	KB-1	4.4(0.55)					
*N*. *rotundifolia*	RG-1	5.0				KM411348	N
*N*. *simulans*	SL19	5.0	2.0(rings)	2.0	4.2(0.45)		
*N*. *simulans*	SL29	5.0	2.2(0.45)	2.2(0.45)	4.8(0.45)	KM411340	N
*N*. *suaveolens*	SL24		2.0	2.0	3.0	KM411306	N
*N*. *truncata*	SL44					KM411342	N
*N*. *umbratica*	Wea-1	3.6(0.55)	1.0	1.0	4.0	KM411303	N
*Nicotiana sp*	BrH-1	5.0				KM411333	N
*Nicotiana sp*	MtG-2	3.0					
*Nicotiana sp*	MtG-4	3.0				KM411350	N
*Nicotiana sp 'Corunna'*	SL23		1.0	1.0	4.0		

The presence (Y) or absence (N) of an insertion/deletion (indel) mutation in the *RDR1* is indicated. GenBank accession codes of partial *RDR1* coding sequences are given.

^a^YTMMV, yellow tailflower mild mottle virus; BYMV, bean yellow mosaic virus: CMV, cucumber mosaic virus; TSWV, tomato spotted wilt virus.

^b^Mean symptom indices (Standard deviation)

0. Systemic infection not detected

1. No visible symptoms of infection.

2. Mild symptoms of chlorosis, mosaic and/or leaf deformation evident. Slight stunting may be evident. Ring patterns or small necrotic spots sometimes visible. Little to no obvious impact on the numbers of flowers or viable seed produced.

3. Moderate symptoms of chlorosis, mosaic and/or leaf deformation. Moderate to significant stunting of growth and small necrotic patches may be present. Some flowers and seed may be produced.

4. Large patches of leaf/stem necrosis, severe stunting. Plant remained alive.

5. The plant was dead.

NL = necrotic lesion; rings = ring patterns

### YTMMV

**Fig 3:**
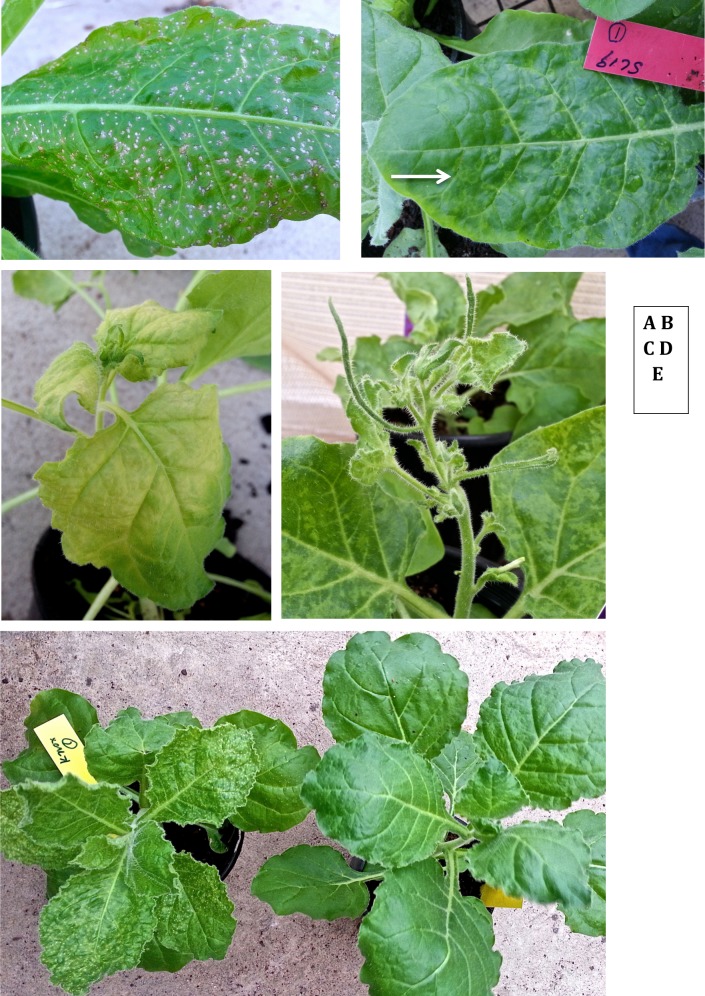
Symptoms of infection. A. Leaf of *N*. *occidentalis ssp hesperis* Nt-5 infected with BYMV exhibiting small necrotic lesions within 7 days of infection. B. *N*. *simulans* SL19 exhibited small chlorotic rings (arrow) 15 dpi with BYMV. C. Symptoms of infection of YTMMV on *N*. *benthamiana* RA-4 20 dpi. D. *N*. *benthamiana* VL552B2.1 exhibiting symptoms of leaf mottling and deformation 20 dpi with YTMMV. E. *N*. *benthamiana* Kx-1 (left) exhibiting chlorotic spots 20 dpi with BYMV. Plant on the right is uninfected.

There were significant differences (p<0.05) among the infected plants by YTMMV. This virus killed members of some of the *Nicotiana* species tested, and it had the highest overall mean symptom severity index, 3.94, of the four viruses tested across all plant accessions (S3 Table, S4 Table). *N*. *benthamiana* laboratory accession RA-4 became wilted and chlorotic within 20 days of infection by YTMMV (Fig 3C), then died between 21 and 35 dpi In contrast, none of the wild accessions of *N*. *benthamiana* died upon infection with YTMMV. Instead, they exhibited moderate symptoms of mosaic and leaf distortion (Table 2, Fig 2B, Fig 3D). The following species and accessions exhibited severe symptoms of disease or died: *N*. *cavicola* SL9, *N*. *excelsior* SL11, all the *N*. *occidentalis* accessions tested, consisting of subspecies *obliqua* and *occidentalis* (accession codes Cl-1, CB-1, SC-2A, 17.3B, SL17, MtA-8, MtA-4, MtA-9, MtA-10, MtA-11, MtA-12, Ft-1, VL552B1.1, UK-4, UK-3, UK-2, UK-1, BC-1, Br-1, TB-1, BC-1) (Fig 2D), the two *N*. *rosulata* accessions tested, consisting of subspecies *ingulba* and *rosulata* (SL18 and SL51, respectively), two accessions of *N*. *simulans* (SL19, SL29), three of *N*. *rotundifolia* (KB-1, HH-1, RG-1), *N*. *umbratica* (Wea-1, Fig 2C), and a *Nicotiana* accession not identified to the species level (BrH-1). Of those that did not die, plants of *N*. *occidentalis* accessions CB-1, Ft-1, and Cl-1, MtA-9, MtA-11, MtA-12, UK-1, UK-3, SC2-A grew new, often chlorotic and deformed shoots from axillary buds, some of which produced flowers and seed. Most of the other species tested exhibited moderate symptoms, the exceptions being accessions of *N*. *heterantha* (Ft-2, Ft-3, SL33) and *N*. *africana* (SL6), which exhibited mild symptoms. *N*. *glutinosa* plants responded with small necrotic local lesions on infected leaves but there was no systemic spread of the virus.

Seeds of *N*. *benthamiana* accessions VL552B2.1, 17.24 and 17.26 were collected from YTMMV-infected parent plants and from three mock-infected plants of the same accessions and germinated. Visual assessment was done at the two-leaf stage of approximately 300 seedlings from each batch. Seedlings derived from infected and uninfected parents appeared indistinguishable and none exhibited symptoms typical of YTMMV infection that were evident on parents. RT-PCR assays using YTMMV-specific primers were carried out RNA extracted from bulked leaf samples from each group of seedlings, and these failed to detect YTMMV.

### BYMV

There were significant differences (p<0.05) among the infected plants by BYMV. All inoculated *Nicotiana* plants became infected, but none of the infected plants died. The range of responses expressed by systemically infected plants was from asymptomatic to severe (symptom indices 1–4) (Table 2, S2 Table, S3 Table, Fig 2A). Most *N*. *benthamiana* accessions tested, including RA-4, responded with similar mild to moderate symptoms. Plants of *N*. *benthamiana* accession Kx-1 and of *N*. *occidentalis* ssp *hesperis* (Nt-1, Nt-4, Nt-5) were unusual in that they exhibited small necrotic lesions on inoculated leaves (Fig 3A and 3E). Plants of *N*. *occidentalis* ssp *hesperis* accessions NT-1, Nt-4 and Nt-5 became severely symptomatic, but *N*. *occidentalis* ssp *obliqua*-VL552B1.1 plants remained mildly symptomatic, and no necrotic lesions were present. Another unusual symptom of BYMV infection was chlorotic ring patterns that occurred on leaves of all infected *N*. *simulans* SL19 plants (Fig 3B), but not on *N*. *simulans* SL29 plants. In plants of severely affected accessions of *N*. *goodspeedii* SL13 and *N*. *heterantha* SL33, the majority of leaves and apical meristems died. These showed signs of recovery when axillary buds emerged by about 35 dpi, but these remained deformed and chlorotic and few flowers, if any, were produced. None of the following exhibited visible symptoms, although they were all systemically infected: *N*. *amplexicaulis* SL7, *N*. *forsteri* SL5, *N*. *excelsior* SL11, *N*. *gossei* SL14, *N*. *rotundifolia* SL20, *N*. *umbratica* Wea-1, *N*. *africana* SL6, and *Nicotiana* species ‘Corunna’ SL23 (Table 2).

### CMV

There were significant differences (p<0.05) among the infected plants by CMV. All *Nicotiana* plants tested became infected with CMV. In most cases, infected plants showed similar patterns of symptom development to those infected with BYMV (the mean difference in overall symptom index between BYMV and CMV was 0.56) (S2 Table). The notable exception was *N*. *occidentalis* ssp *hesperis* Nt-1, where all plants died, whereas the other two *N*. *occidentalis ssp hesperis* accessions exhibited moderate symptoms. The three accessions of *N*. *occidentalis* ssp *hesperis* tested (Nt-1, Nt-4, Nt-5) developed necrotic lesions. *N*. *occidentalis ssp obliqua* SL17 plants were asymptomatic. *N*. *cavicola* SL9 plants that were strongly symptomatic under BYMV infection responded with mild symptoms to CMV infection (Table 2). There were no rings induced by CMV infection on any plant tested.

### TSWV

There were significant differences (p<0.05) among the infected plants by TSWV. All *Nicotiana* plants inoculated became infected, and plants of some accessions were killed by infection with TSWV (symptom index 5), and on all plants tested, overall symptoms of TSWV were more severe (mean symptom index of 3.49) than with BYMV or CMV infection (S2 Table, S3 Table). Generally, plants reacted with symptoms about one category higher on infection with TSWV than they did with BYMV or CMV. Exceptions were plants of *N*. *rotundifolia* SL20 and *Nicotiana* ‘Corunna’ SL23 that had symptoms three categories higher. Infected plants *N*. *occidentalis* ssp *hesperis* Nt-1, Nt-4, Nt-5 and SL15 initially exhibited local necrotic lesions on inoculated leaves and later symptoms were severe or plants died. *N*. *occidentalis* ssp *obliqua* accessions VL552B1.1 and SL17 did not show local lesions on inoculated leaves but later symptoms resembled those of *N*. *occidentalis* ssp *hesperis* accessions. Plants of *N*. *megalosiphon* SL1, *N*. *simulans* SL19 and SL29, *N*. *occidentalis ssp hesperis* SL15, *N*. *cavicola* SL9, *N*. *rotundifolia* SL20, and *Nicotiana* ‘Corunna’ SL23 all exhibited severe symptoms. TSWV did not spread systemically in *N*. *glutinosa*, instead plants responded with small necrotic local lesions on inoculated leaves.

### *RDR1* gene sequence

Fifty-one partial *RDR1* gene fragments were sequenced and GenBank accessions assigned (Table 2), 19 of which were from *N*. *benthamiana* accessions and the rest from accessions of other *Nicotiana* species. Fifty sequences shared >94% nt identity with one another. The notable exception was laboratory accession *N*. *benthamiana* RA-4, which contained the identical 72 nt insertion mutation reported by Yang and colleagues [33]. The wild accessions of *N*. *benthamiana* tested did not contain this insertion or other insertions or deletions or translation stop codons in this gene region, nor did accessions of the other species tested (Table 2).

## Discussion

Responses to infection by YTMMV were significantly different between new wild accessions of *N*. *benthamiana* and laboratory accession RA-4. Although both groups were equally susceptible to infection by YTMMV and to the other viruses tested, as defined by the ability of the virus to replicate and move systemically within the plant, the differences between them were in symptom responses. Every *N*. *benthamiana* RA-4 plant infected with YTMMV was dead by 21–35 dpi, whereas all plants of the new wild accessions responded with moderate symptoms, and none died. Additionally, YTMMV-infected plants of the wild *N*. *benthamiana* accessions produced viable seed. YTMMV was not detected from seedlings grown from seed from three infected *N*. *benthamiana* parent plants, indicating that seed transmission of YTMMV does not occur or it is rare. The insertion mutant *Nb-RDR1m* allele [33] was present in plants of our laboratory accession RA-4, but was absent in all wild *Nicotiana* accessions tested, including all those of *N*. *benthamiana*. It is tempting to speculate that the absence of *Nb-RDR1m* in wild accessions of *N*. *benthamiana* was responsible for preventing systemic necrosis induced by YTMMV infection. Yet, YTMMV-infected plants of *N*. *occidentalis*, *N*. *rosulata*, *N*. *excelsior*, *N*. *forsteri*, and *N*. *cavicola* accessions tested also lacked the mutant *RDR1* allele and all developed symptom indices of 4 or 5, in some cases resembling those observed in *N*. *benthamiana RDR1m* plants. Notably, the response of some *N*. *occidentalis* plants to YTMMV infection initially resembled those observed from *Nb-RDR1m* plants (rapid chlorosis and plant collapse), but later they partially recovered by generating new shoots, and in some cases flowers and seed. The sequences of complete *RDR1* genes were not obtained here, so is conceivable that additional loss-of-function mutations exist in *RDR1* genes of *Nicotiana* species.

Previously, two groups [33, 52] researched the role of *Nb-RDR1m* in response to virus infection. Yang *et al*. [33] used virus-induced gene silencing to show that despite being truncated, *Nb-RDR1m* ameliorates virus-induced (Potato virus X, PVX, *Potexvirus*) symptom development. Both groups attempted to create the equivalent of *N*. *benthamiana Nb-RDR1* plants by complementing the mutant *Nb-RDR1m* with the functional ortholog from another species. Yang *et al*. [33] used the *RDR1* from *Medicago truncatula* (*Mt-RDR1*), creating *Nb-RDR1m* + *Mt-RDR1* (hereafter called *Mt-RDR1* plants) plants, while Ying *et al*. [52] used the *N*. *tabacum Nt-RDR1* to create *Nb-RDR1m* + *Nt-RDR1* (hereafter called *Nt-RDR1* plants) plants. Both groups assumed that a functional transgenic *RDR1* ortholog would be expressed in a dominant manner over the endogenous mutant *Nb-RDR1m*. Ying and colleagues [33] showed that expression of *Nt-RDR1* did not suppress expression of endogenous *Nb-RDR1m*, *Nb-RDR2 or Nb-RDR6* in the transgenic *N*. *benthamiana* lines developed. In the two studies [33, 52], responses to virus infection differed widely. After Cucumber mosaic virus (CMV, *Cucumovirus*), PVX or Potato virus Y (PVY, *Potyvirus*) infection, Yang *et al*. [33] demonstrated that *Mt-RDR1* plants and *Nb-RDR1m* control plants responded similarly in terms of virus accumulation, viral RNA expression, and symptom severity. In contrast, Ying and colleagues [52] found that *Nt-RDR1* plants infected with CMV, PVY and Plum pox virus (*Potyvirus*) displayed more severe symptoms, had higher virus accumulation, and greater viral RNA expression than did *Nb-RDR1m* control plants. Like the earlier study [33], our study showed that infection by the non-tobamoviruses we tested (BYMV, CMV, TSWV) did not induce greater symptom expression in *Nb-RDR1* than in *Nb-RDR1m* plants. When challenged with tobamoviruses, *Mt-RDR1* plants accumulated less TMV, Turnip vein-clearing virus and Sunn hemp mosaic virus, whereas *Nb-RDR1m* control plants were severely symptomatic [33]. Again, the responses reported by Ying and colleagues [52] differed markedly; transgenic *Nt-RDR1* plants responded in a similar manner to *Nb-RDR1m* control plants after infection with TMV and Tomato mosaic virus. Our experiment with *Nb-RDR1m* and *Nb-RDR1* plants and the tobamovirus YTMMV gave results more in line with those of Yang *et al*. [33] than of Ying *et al*. [52]. We found that plants with an apparently functional *Nb-RDR1* were protected against tobamovirus-induced severe symptomology. We are aware that the *Nb-RDR1* and *Nb-RDR1m* plants we used have slightly different genetic backgrounds, so traits other than *RDR1* may be present, and these may also influence symptom response. Other genes known to be involved in antiviral RNA silencing include *DCL2*, *DCL3*, *DCL4*, *DRB4*, *RDR6*, *SGS3*, *HEN1*, *AGO1* and *AGO2* [18, 53], and these were not examined in the current study.

It is unclear why plant responses reported by us and by Yang *et al*. [33] differed so much from those of Ying *et al*. [52]. Possible reasons are the differences observed may relate to the sources of the *RDR1* (*M*. *truncatula* vs *N*. *tabacum* vs *N*. *benthamiana*), or differences in virulence in the virus strains used. The latter explanation does not easily account for the differences seen in CMV symptomology; both Yang *et al*. [33] and Ying *et al*. [52] used genetically similar CMV subgroup I isolates (CMV-Fny and CMV-SD, respectively), whereas in this study we used a relatively dissimilar subgroup II isolate (CMV-SW-11).

Here we determined that there was no difference between *Nb-RDR1* and *Nb-RDR1m* plants in terms of susceptibility to all the viruses tested, as defined by the ability of the virus to infect the plant systemically. Both Yang *et al*. [33] and Ying *et al*. [52] determined that *Mt-RDR1* or *Nt-RDR1* were still susceptible to the tobamoviruses they analysed. Therefore, from all these results it appears that symptom responses and possibly virus titre and viral RNA accumulation, but not susceptibility as such, is associated with *Nb-RDR1* function.

Yang *et al*. [33] proposed that *Nb-RDR1m* is a recent mutation because its nucleotide sequence (other than the 72 nt insertion) closely resembles the *N*. *tabacum Nt-RDR1*, and because *Nb-RDR1m* is still inducible by phytohormone application and virus infection. Our finding that *Nb-RDR1m* was absent in the wild populations we tested tends to support the hypothesis that *Nb-RDR1* is a recent mutation that has not had time to expand its range. On the other hand, it is possible that it was once more widely distributed but now remains in localised populations, perhaps because of tobamovirus epidemics. The sporadic distribution of *N*. *benthamiana* populations in northern Australia may help protect wild *Nb-RDR1m* populations from tobamovirus infection. Tobamoviruses have no known arthropod vectors, but because they have extremely stable particles [54], potentially any creature that eats or contacts them might vector them. The existence of YTMMV, a Solanaceae-infecting tobamovirus, and Clitoria yellow mottle virus, a legume-infecting one [55], makes it possible that other tobamoviruses exist in the Australian flora, and that over time contact with *N*. *benthamiana* populations is likely to occur. Systemic necrosis responses in *Nb*-*RDR1m* plants and in other *Nicotiana* species to YTMMV infection may be examples of ‘field resistance’, where susceptible plants die quickly, thereby removing themselves as sources of infection and slowing virus spread within the population, but we find this scenario improbable because it should lead to the extinction of *Nb-RDR1m*. If the location of the *Nb-RDR1m* population that yielded the original laboratory accession were determined from historical documents, it would be of interest to elucidate natural distribution of *Nb-RDR1m* within it and surrounding populations to establish if its range is expanding or contracting. Similarly, analysis of the dynamics of the acute and persistent viral flora infecting natural *Nb-RDR1m* and *Nb-RDR1* populations would be of great scientific interest in revealing co-evolutionary strategies and ecological roles in natural plant/virus systems.

Ying *et al*. [52] proposed that *Nb-RDR1* has a dual role: that of silencing viral transcripts and of suppressing *Nb-RDR6*-mediated inhibition of systemic virus spread. Their hypothesis that the dysfunctional *Nb-RDR1m* evolved during a prolonged host-virus arms race to favour up-regulation of an *Nb-RDR6*-induced antiviral system seems unlikely given the apparent rarity of *Nb-RDR1m* in the wild, but this hypothesis can be tested by carrying out the distribution and ecology studies of natural *Nb-RDR1m* populations proposed above.

We propose two hypotheses to account for the prevalence in laboratories of the apparently rare-in-the-wild *Nb-RDR1m*. The first hypothesis is that the insertion mutation happened recently in a laboratory during the 70+ years that *N*. *benthamiana* has been utilised by plant biologists. Its discovery in a natural population would immediately discount this hypothesis. The second hypothesis is that seed from a wild *Nb-RDR1m* plant was collected from a natural population, perhaps amongst *Nb-RDR1* plants, and biologists selected the former because of its greater symptom responses to some viruses and perhaps other traits. Indeed, when we compare *Nb-RDR1m* plants with *Nb-RDR1* plants, the former appear to express several unusual traits, some of which may be considered as ‘domestication’ traits. For example, seed from *Nb-RDR1m* plants germinated quickly and evenly 1–3 weeks after harvest, whereas that of wild accessions usually required 6–20 weeks of storage (or GA_4_ treatment) after harvest before it germinated evenly. Uneven physical or physiological seed dormancy is a common survival strategy against desiccation in wild annual plants [56]. The leaves of *Nb-RDR1m* plants are relatively glabrous (hairless), have thin laminas, are paler green, smaller, and with petioles that lacked wings. Thin glabrous laminas enable more efficient virus inoculation. Leaves of wild accessions from inland populations were often covered with hard prickly hairs, had thicker laminas, larger leaf sizes, and sometimes they had winged petioles (e.g accessions MtA 3–7). Leaves from plants collected at coastal sites were glabrous with reflective, rugose, waxy laminas, and without winged petioles (e.g. accession VL552B2.1). Experiencing the same growing conditions under glass, *Nb-RDR1m* plants grew to about a metre in height, whereas plants of some wild accessions (e.g. accession PPM1) grew to two metres. *Nb-RDR1m* plants quickly produced many small white flowers, while wild accessions were generally slower to flower, had fewer but relatively larger flowers (e.g. accession PPM-1), and some were cream to yellow in colour, or tinged with purple pigment. Smaller, faster developing plants are more suited to scientific experiments done in confined spaces. We are cautious to attribute all the unusual traits seen in *N*. *benthamiana* RA-4 plants to human selection. *RDR1* influences small RNA expression, which in turn may influence expression of other genes [57, 58, 59, 60, 61].

The genetic homogeneity of the single available accession of *N*. *benthamiana* has enabled meaningful extrapolation of experiments done in different laboratories over 70 years. The presence of *Nb-RDR1m* has undoubtedly stimulated its acceptance as a model plant for virology and transient gene expression studies. The discovery reported here that the widely used laboratory accession is probably not representative of the species as a whole is surprising and exciting. This finding opens up possibilities of comparisons between accessions, the most obvious being comparative expression analysis of *Nb-RDR1m* and *Nb-RDR1* plants. There appears to be no sexual incompatibility between the few accessions we have already crossed using *N*. *benthamiana*-RA-4 as the female partner, and this is being tested more broadly within the species. Sexual incompatibilities do occur between some accessions of some species, for example *Arabidopsis thaliana* [62, 63].

Any effective biological model system should have available a collection of accessions with a range of genotypical and phenotypical characteristics. For example, *A*. *thaliana*, the most widely used model plant system, has over 300 natural accessions available from across Eurasia [64]. Until recently, *N*. *benthamiana* probably had only one. Although *N*. *occidentalis* has played second fiddle to *N*. *benthamiana* in virus research and expression studies, we envisage that availability of a broader range of subspecies and accessions will enable it too to become a useful model species.

## Supporting Information

S1 Fig A: Marginal means of symptom severity induced in 15 *Nicotiana* accessions systemically infected with four viruses (Tables 1, 2). Symptom indices of 0–5 represent a range of responses to inoculation from (1) systemic infected detected but no symptoms observed to (5) systemic infection detected leading to whole plant death. B: Overall comparison of the viruses assessed using marginal means of symptom severity induced in 15 *Nicotiana* accessions.
(TIF)

S1 Table Virus specific primer sequences (5’ > 3’) used to confirm infection by Yellow tailflower mild mottle virus (YTMMV), Bean yellow mosaic virus (BYMV), Cucumber mosaic virus (CMV), and Tomato spotted wilt virus (TSWV). (DOCX)

S2 Table Sequences (5’ > 3’) of primers used to amplify part of the *Nicotiana RDR1* gene. Numbers in parentheses represent the annealing coordinates of the primers on the *N*. *benthamiana Nb-RDR1m* sequence (GenBank accession AY574374).
(DOCX)

S3 Table Comparison symptom severity induced by four viruses on 75 *Nicotiana* plants using severity of symptoms as the dependent variable. This is a measure of relative symptom severity induced by each virus. Thus, mean differences >1 indicates overall greater symptom severity is induced by virus I than by virus J, while mean differences <1 indicates overall milder symptoms induced by virus I compared to virus J.
(DOCX)

S4 Table Overall symptom severity indices of four viruses infecting 75 *Nicotiana* plants (five plants each of 15 different *Nicotiana* accessions) as calculated by Tukey B analysis. Symptom indices of 0–5 represent a range of responses to inoculation from (1) systemic infected detected but no symptoms observed to (5) systemic infection detected leading to whole plant death. Thus, as indices of severity increase, symptom severity increases.
(DOCX)
