# Supplementary figures and images for: Differential Responses to Virus Challenge of Laboratory and Wild Accessions of Australian Species of Nicotiana, and Comparative Analysis of RDR1 Gene Sequences
Source: PLoS One. 2015 Mar 30;10(3):e0121787. doi: 10.1371/journal.pone.0121787 (PMC4379023; doi:10.1371/journal.pone.0121787)

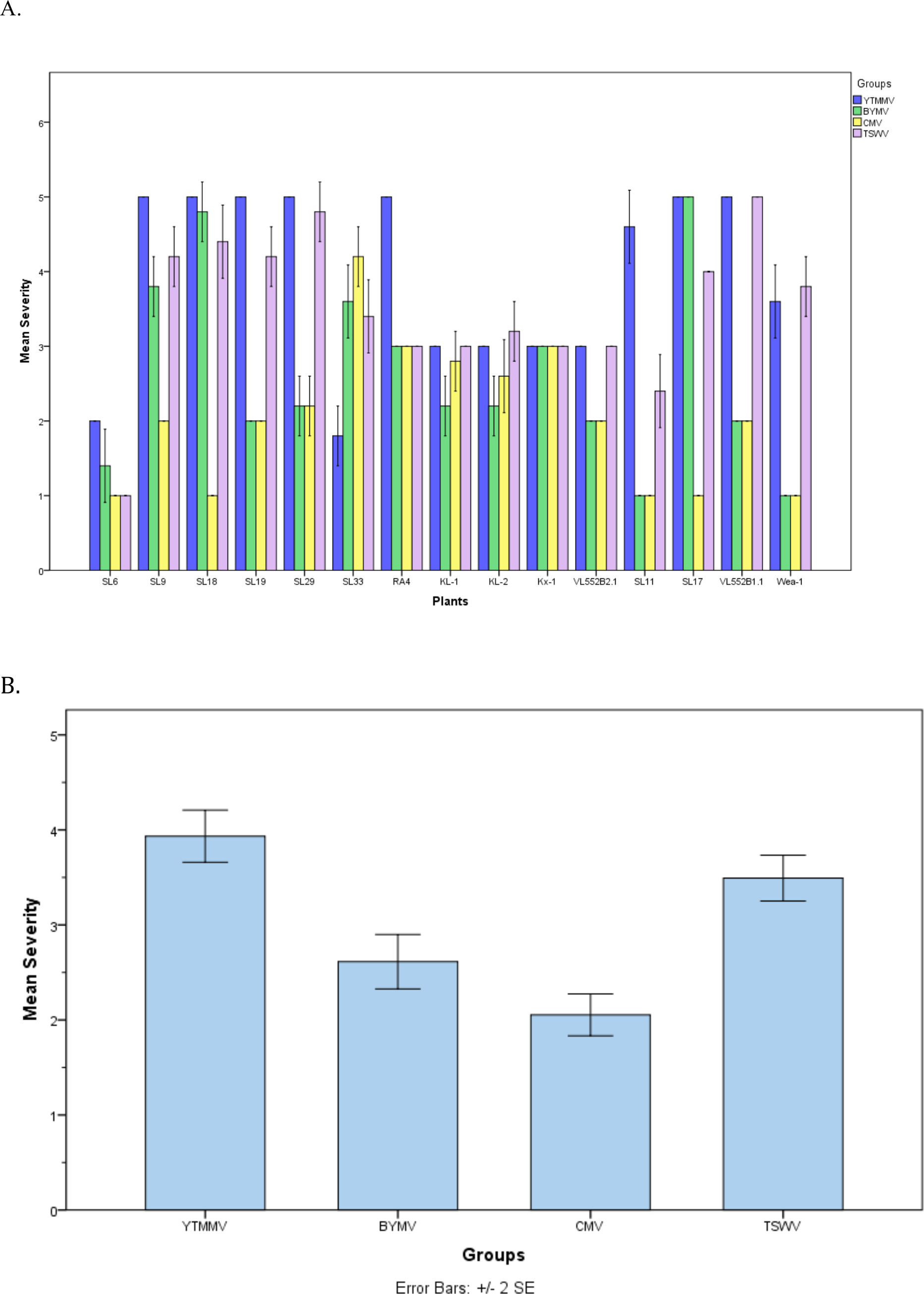

Supplement: S1 Fig — Symptom indices of 0–5 represent a range of responses to inoculation from (1) systemic infected detected but no symptoms observed to (5) systemic infection detected leading to whole plant death. B: Overall comparison of the viruses assessed using marginal means of symptom severity induced in 15 Nicotiana accessions. (TIF) [file pone.0121787.s001.tif]
