# Supplementary material for: Differential Responses to Virus Challenge of Laboratory and Wild Accessions of Australian Species of Nicotiana, and Comparative Analysis of RDR1 Gene Sequences
Source: PLoS One. 2015 Mar 30;10(3):e0121787. doi: 10.1371/journal.pone.0121787 (PMC4379023; doi:10.1371/journal.pone.0121787)
Supplement: S1 Table — (DOCX) [file pone.0121787.s002.docx]

| **Virus and primer names (F and R)** | **Forward Primer** | **Reverse Primer** |
| --- | --- | --- |
| YTMMV461F, YTMMV809R | GATGTTCGTGACGTCATGCG | TAGCGGGTAACTCCACGGTA |
| BYMV13, BYMV14a | GGGCATTGGAATAAACCAGT | TAGGCTATGGATTCCCACTA |
| CMV1, CMV2 | GCCGTAAGCTGGATGGACAA | TATGATAAGAARCTTGTTTCGCG |
| TSWV. Primer-A, Primer-B | CCTTCCTCTTCCTCTTCAACTG | TCTGCCCCACTATACCAAACC |
