# Supplementary material for: Differential Responses to Virus Challenge of Laboratory and Wild Accessions of Australian Species of Nicotiana, and Comparative Analysis of RDR1 Gene Sequences
Source: PLoS One. 2015 Mar 30;10(3):e0121787. doi: 10.1371/journal.pone.0121787 (PMC4379023; doi:10.1371/journal.pone.0121787)
Supplement: S2 Table — Numbers in parentheses represent the annealing coordinates of the primers on the N. benthamiana Nb-RDR1m sequence (GenBank accession AY574374). (DOCX) [file pone.0121787.s003.docx]

| **Primer pair (F and R)** | **Forward Primer** | **Reverse Primer** |
| --- | --- | --- |
| RP1, RP2 | CACCATGCAAAGTTTATTTTTGTGGTCCAGA (1171-1201) | GCCCGGAAAGTTTGCAGCATCATTGAAAGAAA (2178 – 2209) |
| RP120614, RP220614 | TTTCTTGCATTTTCATCGAGCCAGTTG (1413-1439) | GTCASCAGATATTTTWCCAATTCCATCAGAAAAG (1730-1763) |
| RP1new, RP2new | TTTCTTGCATTTTCATCGAGC (1413-1433) | CTATTTCTCAAAGATAACTTCATT (1874-1897) |
