# Supplementary material for: Differential Responses to Virus Challenge of Laboratory and Wild Accessions of Australian Species of Nicotiana, and Comparative Analysis of RDR1 Gene Sequences
Source: PLoS One. 2015 Mar 30;10(3):e0121787. doi: 10.1371/journal.pone.0121787 (PMC4379023; doi:10.1371/journal.pone.0121787)
Supplement: S3 Table — This is a measure of relative symptom severity induced by each virus. Thus, mean differences >1 indicates overall greater symptom severity is induced by virus I than by virus J, while mean differences <1 indicates overall milder symptoms induced by virus I compared to virus J. (DOCX) [file pone.0121787.s004.docx]

| (I) Virus | (J) Virus | Mean Difference (I-J) | Std. Error | Sig. | 95% Confidence Interval | |
| --- | --- | --- | --- | --- | --- | --- |
|  |  |  |  |  | Lower Bound | Upper Bound |
| YTMMV | BYMV | 1.32^*^ | .048 | .000 | 1.19 | 1.45 |
|  | CMV | 1.88^*^ | .048 | .000 | 1.75 | 2.01 |
|  | TSWV | .44^*^ | .048 | .000 | .31 | .57 |
| BYMV | YTMMV | -1.32^*^ | .048 | .000 | -1.45 | -1.19 |
|  | CMV | .56^*^ | .048 | .000 | .43 | .69 |
|  | TSWV | -.88^*^ | .048 | .000 | -1.01 | -.75 |
| CMV | YTMMV | -1.88^*^ | .048 | .000 | -2.01 | -1.75 |
|  | BYMV | -.56^*^ | .048 | .000 | -.69 | -.43 |
|  | TSWV | -1.44^*^ | .048 | .000 | -1.57 | -1.31 |
| TSWV | YTMMV | -.44^*^ | .048 | .000 | -.57 | -.31 |
|  | BYMV | .88^*^ | .048 | .000 | .75 | 1.01 |
|  | CMV | 1.44^*^ | .048 | .000 | 1.31 | 1.57 |

Based on observed means

Error term is Mean Square (Error) = .087.

*The mean difference is significant at the .05 level.
