# Supplementary material for: Differential Responses to Virus Challenge of Laboratory and Wild Accessions of Australian Species of Nicotiana, and Comparative Analysis of RDR1 Gene Sequences
Source: PLoS One. 2015 Mar 30;10(3):e0121787. doi: 10.1371/journal.pone.0121787 (PMC4379023; doi:10.1371/journal.pone.0121787)
Supplement: S4 Table — Symptom indices of 0–5 represent a range of responses to inoculation from (1) systemic infected detected but no symptoms observed to (5) systemic infection detected leading to whole plant death. Thus, as indices of severity increase, symptom severity increases. (DOCX) [file pone.0121787.s005.docx]

| **Severity** | | | | | | |
| --- | --- | --- | --- | --- | --- | --- |
|  | Virus | N | Subset | | | |
|  |  |  | 1 | 2 | 3 | 4 |
| Tukey B^a,b^ | CMV | 75 | 2.05 |  |  |  |
|  | BYMV | 75 |  | 2.61 |  |  |
|  | TSWV | 75 |  |  | 3.49 |  |
|  | YTMMV | 75 |  |  |  | 3.93 |

Observed means for viruses in homogeneous subsets are displayed.

Error term is Mean Square (Error) = 0.087

1. Harmonic Mean Sample Size = 75
2. Alpha = 0.05
